# Hemodynamic assessment of intracranial atherosclerotic stenosis: comparison between invasive non-hyperemic pressure ratio and angiography-derived quantitative flow ratio

**DOI:** 10.3389/fneur.2024.1466864

**Published:** 2024-11-13

**Authors:** Xiaohui Wang, Yang Bian, Rongju Zhang, Haojing Zhu, Junjie Yang, Ruiling Wang, Xinfeng Liu, Bin Lv, Xiangyu Cao, Wei Dai, Zhibin Sun, Jing Jing, Zhihua Du, Shengyuan Yu, Jun Wang

**Affiliations:** ^1^Department of Neurology, The First Medical Center, Chinese PLA General Hospital, Beijing, China; ^2^Health One Departments, The Second Medical Center, Chinese PLA General Hospital, Beijing, China; ^3^Department of Cardiology, The First Medical Center, Chinese PLA General Hospital, Beijing, China; ^4^Department of Clinical Laboratory, The Third Medical Center, Chinese PLA General Hospital, Beijing, China; ^5^Department of Mathematics, College of Science, Hebei Agricultural University, Baoding, China

**Keywords:** intracranial atherosclerotic stenosis, hemodynamics, pressure wire, cerebrovascular pressure ratio, quantitative flow ratio, full-cycle ratio

## Abstract

**Objectives:**

To evaluate the diagnostic accuracy of the quantitative flow ratio (QFR) for hemodynamic exploration of intracranial atherosclerotic stenosis, using the invasive cerebrovascular pressure ratio (CVPR) and resting full-cycle ratio (RFR) as reference standards.

**Materials and methods:**

Patients with symptomatic unifocal intracranial atherosclerotic stenosis were included. The CVPR was defined as the ratio of the proximal and distal pressures. All patients underwent angioplasty under general anesthesia. The QFR was calculated based on digital subtraction angiography. Using the CVPR as a reference, we compared its correlation with the QFR across different degrees and locations of stenosis.

**Results:**

The CVPR and QFR were measured in 34 vessels of 32 patients. The QFR demonstrated a high correlation and excellent agreement(*r* = 0.8227, *p* < 0.001) with the CVPR in distal stenosis before intervention. In the subgroup with diameter stenosis >80%, the QFR showed a high correlation (*r* = 0.8812, *p* < 0.001) with the CVPR. In the anterior circulation subgroup, the QFR showed an excellent correlation (*r* = 0.9066, *p* < 0.001) with the CVPR. In the posterior circulation subgroup, the QFR showed a high correlation with the CVPR (*r* = 0.7706, *p* < 0.001). Diameter stenosis rates showed a moderate negative correlation with the CVPR.

**Conclusion:**

There was a strong correlation between the QFR and wire-based CVPR, especially for anterior circulation lesions before intervention. The QFR may serve as a predictive factor for evaluating hemodynamic changes in intracranial atherosclerotic stenosis.

## 1 Introduction

Intracranial atherosclerotic stenosis (ICAS) is the most common reason of ischemic stroke worldwide, especially in Asian populations, with a higher incidence than that in other ethnic groups. In Asian populations, ICAS accounts for approximately 30–54% of ischemic cerebral infarctions and transient ischemic attacks (TIAs) compared with 5–10% in White populations and 15–29% in Black populations (1–5). The risk of recurrent stroke and death is increased by 25–30% in symptomatic intracranial arterial stenosis (6). In China, the morbidity and mortality of stroke remain high throughout the year (7). The recurrence rate 30 days after cerebral infarction in patients with intracranial artery stenosis is up to 15% after active drug treatment (8).

Therefore, it is important to provide patients with better evaluation, optimized screening, and accurate identification. In addition to the degree of artery stenosis and collateral compensation, hemodynamic estimation is vital for ICAS. At present, the clinical evaluation of blood flow is mainly related to perfusion examinations, including computed tomography perfusion (CTP) examination and perfusion-weighted magnetic resonance imaging. These examinations play important roles in facilitating hemodynamic evaluation and enhancing the efficacy of percutaneous transluminal angioplasty and stenting for intracranial artery stenosis, particularly of the middle cerebral artery (9, 10). However, the utility of CTP in the assessment of the posterior circulation is limited, and one study has shown that CTP has limited diagnostic utility in cases involving acute ischemia of the posterior circulation (11). For cases with bilateral lesions in the intracranial arteries, the accuracy of these perfusion tests may be affected by the lack of good contrast, precluding direct assessment of cerebral hemodynamics in ICAS. To address these limitations, Miao et al. (12) and Liu et al. (13) evaluated changes in cerebrovascular hemodynamics in patients with ICAS using a pressure guidewire in the cerebral vasculature, which verified its safety and feasibility. No serious adverse events associated with the device or procedure occurred.

Fractional flow reserve (FFR) using an invasive pressure guidewire has become one of the gold standards for evaluating hemodynamic deficiency of coronary artery stenosis (14). Studies have shown that resting indices derived from the pressure measurements at rest, without the administration of adenosine, are also independent measures of ischemia (15, 16), thereby reducing the need for hyperemic agents that could cause adverse reactions. In fact, some validation studies have demonstrated that non-hyperemic pressure ratios (NHPRs), including the resting full-cycle ratio (RFR) and instantaneous wave-free ratio (iFR), have similar diagnostic performance to FFR in identifying ischemia-causing coronary lesions (17, 18). The RFR is a non-hyperemic pressure-derived index based on unbiased identification of the lowest distal arterial pressure (Pd)/proximal arterial pressure (Pa) within the entire cardiac cycle and has been shown to be equivalent to the iFR in clinical practice (19). However, hemodynamic assessments of cerebrovascular intravascular pressure are currently lacking. In this regard, some studies used a pressure guidewire to measure the Pa and Pd of intracranial arterial stenosis, and geometric directories based on angiography were correlated with pressure gradient indices between the proximal and distal ends of the stenosis (20).

The quantitative flow ratio (QFR) is a novel method for estimating the fractional flow rate using computational fluid dynamics computations of the coronary arteries from the angiograph. This measurement does not require hyperemic agents or invasive pressure wires, and can virtually evaluate FFR (21). Previous studies from China, Europe, and Japan have verified the feasibility and accuracy of QFR assessment in determining the hemodynamic significance of coronary stenoses, in contrast to FFR measurement using invasive pressure wires (22, 23). However, there is a lack of relevant comparative cerebrovascular studies to verify their accuracy. Therefore, this study aimed to evaluate the diagnostic accuracy of QFR for hemodynamic exploration of ICAS, using the invasive cerebrovascular pressure ratio (CVPR) and RFR as reference standards, in which the pressure ratio between the distal and proximal of the stenosis is different from FFR, because the intracranial artery is far from the heart, the pressure ratio is not equivalent to the flow ratio. So we defined the measured ratio as CVPR (the ratio of the proximal and distal pressure), which is also termed as FF (fractional flow) in the relevant literature.

## 2 Materials and methods

### 2.1 Study design and patients

This study enrolled patients with ICAS who underwent cerebral angiography and NHPR measurements using a pressure guidewire between February 2021 and April 2022. The inclusion criteria were as follows: (1) age 18 to 80 years, (2) presence of recurrent TIA or ischemic stroke within 6 months, (3) stenosis rate of 50–99% confirmed by cerebral angiography, (4) onset of TIA or ischemic stroke symptoms >21 days, and (5) modified Rankin Scale (mRS) score ≤ 3. The exclusion criteria were as follows: (1) tandem lesion with concurrent stenosis or occlusion; (2) any form of intracranial hemorrhage within 3 months; (3) severe coronary atherosclerotic cardiopathy intolerance under general anesthesia; and (4) patients with intracranial hydrocephalus, arteriovenous malformation, aneurysm, or tumor. This study was approved by the Ethics Committee of Chinese PLA General Hospital, and patients or their legally authorized representative provided written informed consent. To limit the effect that the degree of stenosis might have on collateral compensation, all patients were classified into subgroups based on percentage diameter stenosis (DS%) by cerebral angiography: DS% ≤ 80% subgroup and DS% > 80% subgroup.

### 2.2 Cerebrovascular angiography and measurement of invasive NHPRs

General anesthesia was administered to all patients. Access was established through conventional neurointerventional surgery for ICAS, and an appropriate type of intermediate catheter or guide catheter was advanced to the proximal end of the target artery. A microcatheter was used to guide a 0.014-inch pressure wire (C12008; Abbot St. Jude Medical, Minneapolis, MN, United States) to measure the intracranial pressure of the proximal and distal lesions of the stenosis. CVPR and RFR were calculated without hyperemia.

Pd was defined as the pressure at the distal end of the stenosis and Pa was the pressure on the intermediate catheter; both were measured. The CVPR and RFR were measured and calculated automatically using the Abbott Vascular instrument with QUANTIEN™ System (C12787; St. Jude Medical, Minnesota, United States). The CVPR, a parameter close to the FFR, was calculated as Pd/Pa. RFR was defined as the point with the lowest Pd/Pa ratio during the whole cardiac cycle. RFR was calculated by instantaneous Pd/Pa measuring continuously around five cardiac cycles.

### 2.3 Quantitative flow ratio assessment

**Figure 1:**
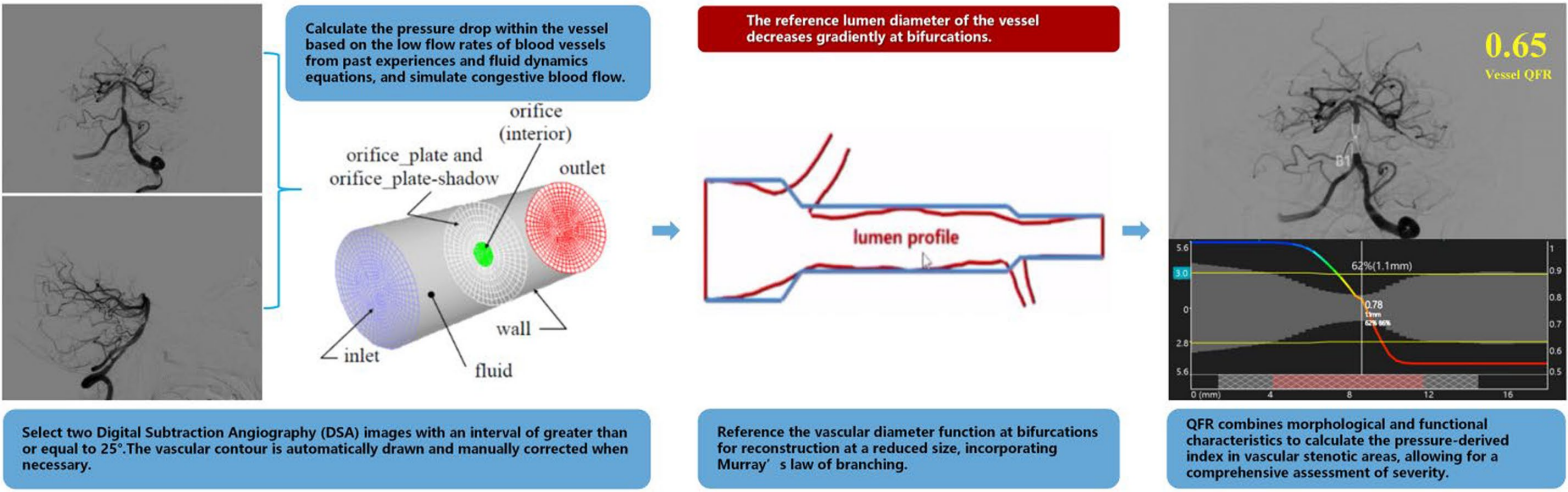
Schematic of QFR calculation process.

The calculation of the QFR was performed using a prototype software by Pulse Medical Imaging Technology made in Shanghai (AngioPlus Core) by an experienced analyst blind to the QFR data (24). The following steps were primarily followed for the computation. (a) Two image projections of digital subtraction angiography (DSA) were selected for analysis of the primary vessel and associated lateral branches, acquired greater than or equal to 25° apart and displayed presenting the minimum overlap and least stenosis. (b) Contour lines were drawn automatically and manually corrected when appropriate. The key frames with a clear outline were selected as the analysis frames, with side branch diameters of ≥1.0 mm and a clear outline at the narrow segment. (c) The reference diameter function was reconstructed with a reduced size at the bifurcations, and performed according to the Murray fractal law (25). (d) The hyperemic flow velocity was modeled based on the pressure drop calculation from the empirical low velocity and the hydrodynamic equation (26), assuming a blood density of 1,060 kg/m^3^ and viscosity of 0.0035 kg/(ms) (27). In addition, QFR was calculated from the mean empirical flow velocities of the intracranial arteries (0.60 m/s for middle cerebral artery and 0.40 m/s for vertebrobasilar artery and internal carotid artery) (28). QFR combines morphological and functional features to calculate a pressure-derived index across the stenosis area, allowing for a comprehensive assessment of severity (Figure 1).

### 2.4 Statistical analysis

The statistical analyses were completed using SPSS version 26.0, Python of Anaconda Navigator (version 1.9.7, Anaconda), and GraphPad Prism 9 (Software, California, Belgium). Correlation analyses between QFR, RFR, and CVPR were performed using the Pearson correlation coefficient method. The goodness of fit $R^{2}$, the correlation coefficient (r), and *p*-value were used as important indicators to evaluate linear correlation. The Bland–Altman plot was used to assess the consistency of the two continuous variable measurements. Statistical significance was defined as *p* < 0.05.

As there is no recognized critical value for CVPR in ICAS, two empirical critical values based on the FFR threshold for coronary heart disease (CVPR = 0.75, CVPR = 0.80) were established to investigate the diagnostic efficiency of QFR and the percentage diameter of stenosis based on the value of coronary artery stenosis. Computation with the area under the curve (AUC) of the receiver operating characteristic was to assess the predictive accuracy.

## 3 Results

In this study, 32 patients with 34 cerebrovascular lesions were included. A single lesion was examined in 30 patients, and two lesions examined in two patients.

### 3.1 Baseline clinical characteristics

The average patient age was 58.7 ± 6.2 years; 29 patients were male, 37.5% were diabetic, and 84.4% were suffering from new ischemic stroke or TIA. There were 12 patients with anterior circulation lesions (nine in the intracranial segment of the internal carotid artery and three in the middle cerebral artery) and 22 with posterior circulation lesions (eight in the intracranial segment of the vertebral artery and 14 in the basilar artery; Table 1). All patients underwent percutaneous transluminal angioplasty consisting of balloon dilatation alone or with stenting, and CVPR measurement before and after interventional surgery.

**Table 1:** Characteristics of the patients at baseline.

Characteristic	*N* = 32
Age, mean (±SD), years	58.7 ± 6.2
Male sex, *n* (%)	29 (90.6)
Body-mass index	26.3 ± 2.9
Diabetes mellitus, *n* (%)	10 (31.3)
Hypertension, *n* (%)	24 (75)
Dyslipidemia, *n* (%)	12 (37.5)
Smoking status, *n* (%)	
Current smoker	14 (43.7)
Former smoker	7 (21.9)
Never smoked	11 (34.4)
Coronary heart disease, *n* (%)	6 (18.8)
Previous PCI, *n* (%)	4 (12.5)
Qualifying event-stroke, *n* (%)	12 (37.5)
Qualifying event-TIA, *n* (%)	15 (46.9)
Lesion vessel, *n* (%)	
Intracranial segment of internal carotid artery	9 (26.5)
Intracranial segment of vertebral artery	8 (23.5)
Basilar artery	14 (41.2)
Middle cerebral artery	3 (8.8)

**Figure 2:**
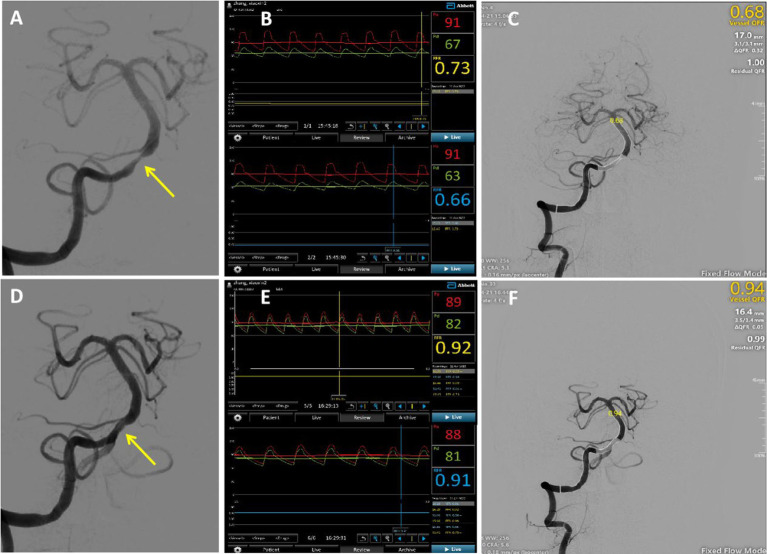
Typical case: A 52-year-old male was admitted due to intermittent dizziness with blurred vision in the right eye for 20 days. Panel (A) showed severe basilar artery stenosis (yellow arrow) before surgery; (B) the CVPR and RFR values of the distal stenosis measured using an invasive pressure wire were 0.73 and 0.66; and (C) the calculated QFRwas 0.68 in the same lesion. The bottom row presented the postoperative figure, and panel (D) showed an obvious improvement in stenosis; (E) the CVPR and RFR values were increased to 0.92 and 0.91; and (F) QFR was noted to have recovered significantly to 0.94.

An illustrative example is shown in Figure 2. A 52-year-old man was diagnosed with a TIA and severe basilar artery stenosis. Cerebral angiography revealed a severe lesion in the basilar artery (Figure 2A). In the distal stenosis lesion, the CVPR and RFR measured by invasive pressure wire were 0.73 and 0.66, respectively (Figure 2B). QFR measured at the same location was 0.68 (Figure 2C), with a clear pressure ratio decrease. Post basilar artery angioplasty, the stenosis was significantly improved (Figure 2D). The CVPR and RFR values increased to 0.92 and 0.91 (Figure 2E), and the QFR value recovered significantly to 0.94 (Figure 2F).

### 3.2 Correlation between quantitative flow ratios and cerebrovascular pressure ratios

**Figure 3:**
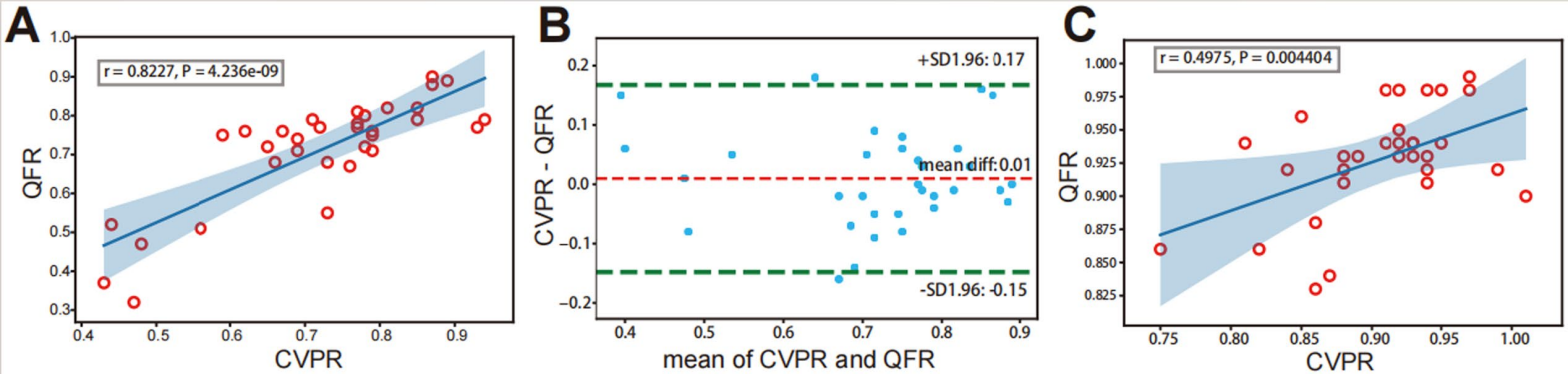
Correlation and consistency between QFR and CVPR. Before interventional surgery, strongly positive correlation (A) and agreement (B) is observed between QFR and CVPR. (C) After interventional surgery, a weakly positive correlation is observed between QFR and CVPR. The light blue area represents predicted “QFR” for a confidence interval of 99%.

**Figure 4:**
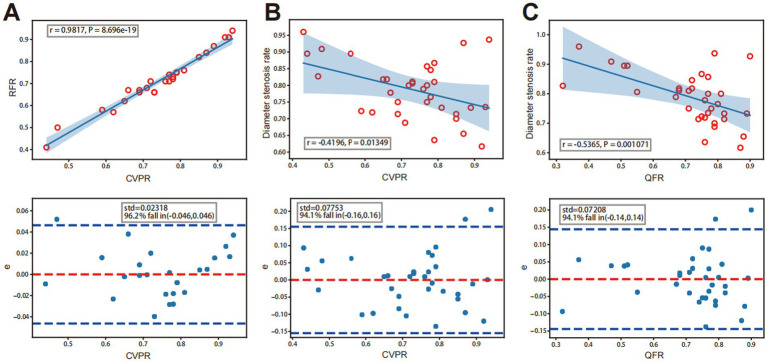
A strong, positive correlation between CVPR and RFR. Analysis diagram of a linear correlation is in the figure above, and the figure below is the residual error diagram. (A) Correlation between QFR or CVPR and diameter stenosis rate. (B) A weekly negative correlation between CVPR and DS%. (C) Medium negative correlation between QFR and DS%.

In the preoperative evaluation of intracranial atherosclerotic stenosis, QFR showed a high correlation (*r* = 0.8227, *p* < 0.001) with CVPR (Figure 3A). Prominent consistency was demonstrated between CVPR and QFR (95% limits of agreement: −0.15 to 0.17, Figure 3B). After interventional surgery, QFR revealed a moderate correlation (*r* = 0.4975, *p* = 0.004) with CVPR (Figure 3C). RFR showed an excellent correlation (*r* = 0.9817, *p* < 0.001) with CVPR, the difference around the fitted regression line was distributed by residual plot presents (Standard Deviation: (SD) = 0.02318, Figure 4A).

#### 3.2.1 Subgroup analysis based on different vessels and degree of stenosis

**Figure 5:**
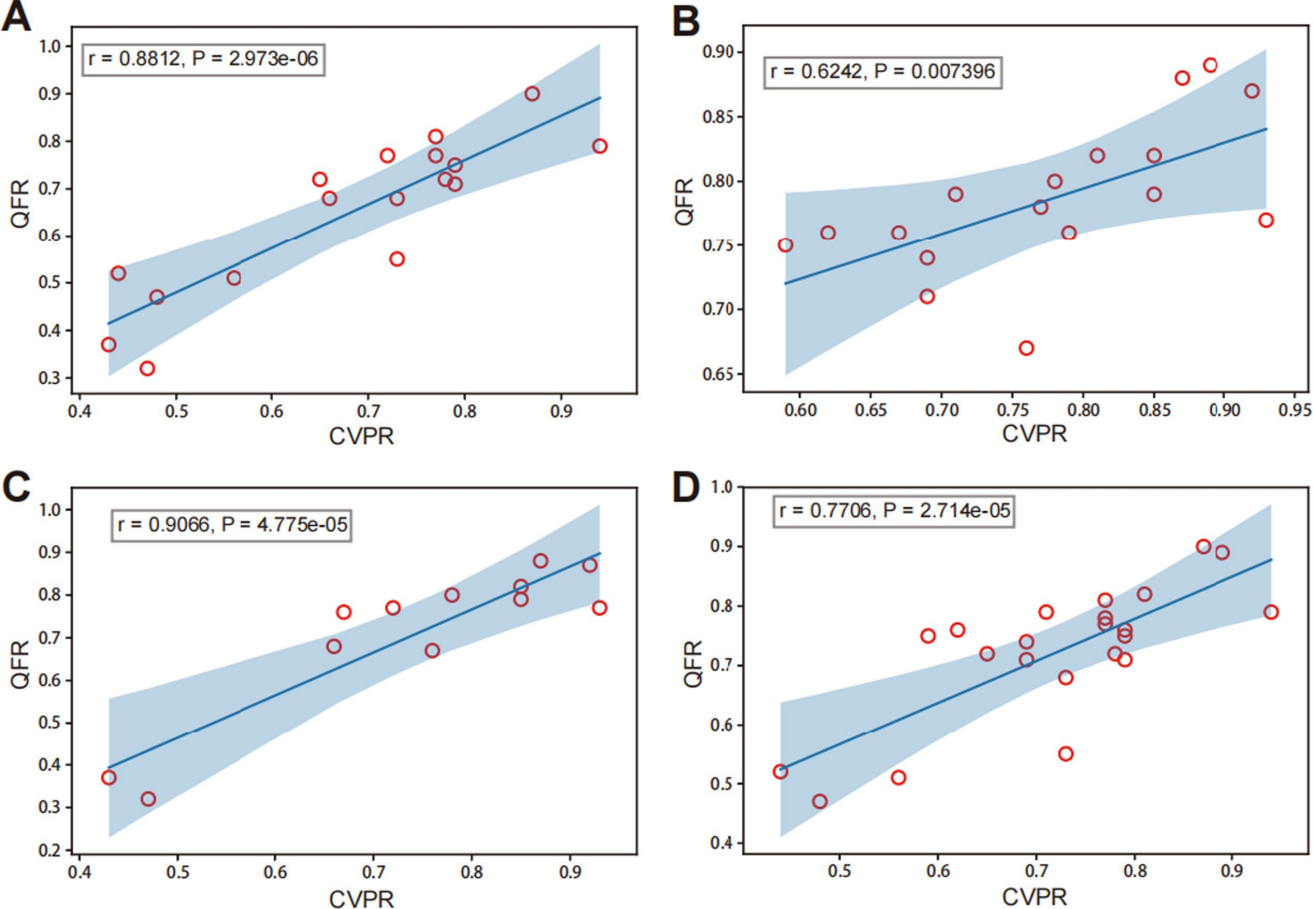
Correlation analysis of QFR and CVPR among different subgroups. Panels (A) and (B) present different degrees of diameter stenosis (DS): (A) DS% >80 and (B) DS% ≤80%. Panels (C) and (D) represent different lesions: (C) the anterior circulation (middle cerebral artery and internal carotid artery in intracranial segment) and (D) posterior circulation (basilar artery and vertebral artery in intracranial segment).

Among groups categorized by degrees of DS, the DS% >80 subgroup showed a high correlation between QFR and CVPR (*r* = 0.8812, *p* < 0.001; Figure 5A). The DS% ≤80% subgroup showed a moderate correlation between QFR and CVPR (*r* = 0.6242, *p* = 0.007; Figure 5B). Among the different vessel lesions groups, the anterior circulation subgroup showed an excellent correlation between QFR and CVPR (*r* = 0.9066, *p* < 0.001; Figure 5C). The posterior circulation subgroup showed a high correlation between QFR and CVPR (*r* = 0.7706, *p* < 0.001; Figure 5D).

#### 3.2.2 Subgroup analysis between DS% and CVPR and QFR

DS rates showed a moderate inverse correlation with CVPR (*r* = −0.4196, *p* = 0.013), and the difference around the fitted regression line was distributed by residual plot (SD = 0.07753; Figure 4B). DS rates showed a moderate negative correlation with QFR (r = −0.5365, *p* = 0.001) and the residual plot around the fitted regression line (SD = 0.07208; Figure 4C).

### 3.3 Diagnostic efficiency of QFR

**Figure 6:**
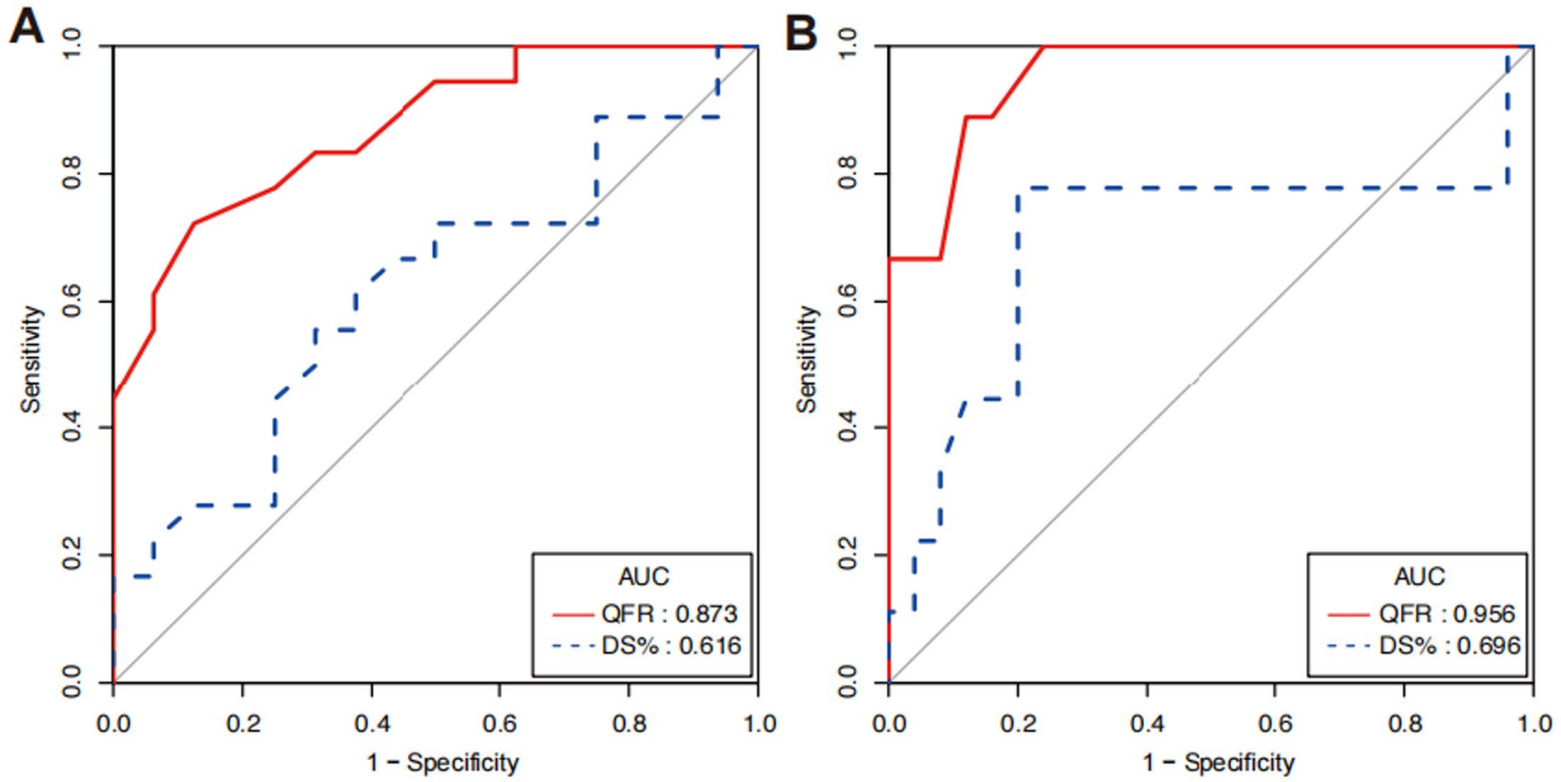
Contrast of diagnostic efficiency between QFR and DS% by ROC analyses. (A) ROC curves for QFR and DS% when CVPR <0.75 and (B) ROC curves when CVPR <0.8.

Regarding the cut-off quantitative value of coronary artery lesions, a CVPR value of <0.75 was considered to cause significant hemodynamic disturbance. The area under the curve for QFR was higher than that for DS% (0.873, 95% CI 0.759 to 0.988 vs. 0.616, 95% CI 0.422 to 0.811, *p* = 0.004), indicating QFR has significantly higher diagnostic efficiency than DS% (Figure 6A). The results were obtained when assuming a CVPR of <0.80 as the critical value (0.956, 95% CI 0.894 to 0.999 vs. 0.696, 95% CI 0.435 to 0.956, *p* = 0.051; Figure 6B), which showed no statistical significance, but a trend that QFR can better reflect the degree of hemodynamic disturbance was observed.

## 4 Discussion

In this study, we adapted the calculation method of the coronary FFR to measure intracranial atherosclerotic stenosis lesions using NHPRs, including CVPR and RFR. In some studies on coronary stenosis (29), NHPRs, such as the ratio at rest in coronary stenosis in the distal to proximal coronary pressure (Pd/Pa), were more accurate than the FFR. Compared with coronary FFR, CVPR is not completely representative of the fractional flow in cerebrovascular diseases. CVPR measurements can only represent pressure values at different positions of the intracranial artery, and the change in vascular pressure is an important hemodynamicparameters. Hemodynamic indicators have garnered increasing attention when compared to DS% and clinical symptoms. Cerebral perfusion examinations, although commonly employed in clinical practice, can be adversely influenced by various subjective and objective factors. For instance, Transcranial Doppler (TCD) requires a high level of professional expertise from inspectors, while CTP or PWI may not accurately evaluate hemodynamic changes in bilateral lesions. Furthermore, these methods demonstrate poor accuracy in assessing perfusion within the posterior circulation lesions due to the complexity of rich autoregulation and collateral circulation (30). Thus, we contend that direct pressure measurement upstream and downstream the stenosis serves as the most intuitive representation and an indicator of hemodynamic abnormalities in the target vessel.

### 4.1 Circulation further complicate the cerebral hemodynamic status

In previous studies on coronary artery disease, the agreement and correlation between the QFR and FFR have been well verified, and QFR has become widely used for guiding coronary intervention operations (31). In the cerebrovascular field of intracranial atherosclerotic stenosis, few studies have compared QFR and invasive pressure ratio measurements. The classical QFR calculation is widely used for assessing coronary artery stenosis, but there is a notable shortage of related calculations in the field of cerebrovascular disease. Measurement of the QFR in the intracranial arteries is still being explored. The new QFR calculation method considers the side branches that are closer to natural bifurcation physiology. Instead of assuming linear tapering, a function of step-down reference diameter using Murray bifurcation fractal law is used to reconstruct more accurate reference vessel dimensions (24). The QFR calculation in this paper has been modified to account for the blood flow velocity of cerebral arteries, which may introduce certain limitations. Our team is attempting to optimize the measurements based on 3D-DSA images and artificial intelligence calculations, aiming to find a more suitable measurement method for intracranial arteries.

The measurement and calculation of CVPR and QFR were completed before and after surgery, which more comprehensively covered the different vascular conditions in our study. Most lesions demonstrated severe stenosis before surgery, whereas, after surgery, mild or no stenosis was observed. The results showed a good degree of agreement and correlation between non-invasive QFR and invasive CVPR, especially preoperatively, when the correlation of QFR and CVPR was more significant. Compared with CVPR measurement, non-invasive QFR measurement has more advantages in clinical application, as patients with CVPR measurement require general anesthesia, and a pressure guidewire in the intracranial artery increases the probability of surgical risk. QFR measurement could be used to calculate the pressure ratio of the distal stenosis lesion with high reliability based on cerebral angiography, determining whether stenosis leads to hemodynamic variation. The analysis after the interventional operation of ICAS showed that the correlation between QFR and CVPR was significantly reduced, which might be related to intima damage in the target arterial lumen and the different extents of dissection that appeared during the operation. Although the level of stenosis was obviously improved, it was repaired slowly with a topical vascular wall; therefore, QFR could not completely represent hemodynamic alterations.

In addition, collateral compensation of the intracranial arteries was considered one of the factors affecting the flow in stenotic lesions. The more severe the stenosis, the more obvious the existence of collateral compensation (32, 33). Therefore, a subgroup analysis was performed according to different degrees of stenosis, with >80% defined as absolute severe stenosis and 60–80% as possible severe stenosis. In the subgroup with stenosis >80%, the R-value was 0.8812 in the correlation comparison between QFR and CVPR, while in the group with <80% stenosis, it was 0.6242, indicating that the correlation between QFR and CVPR was stronger in patients with more severe stenosis. This indicates that the more severe the stenosis, the stronger the correlation between QFR and CVPR, which might be a higher reference for clinical impact.

We also performed a subgroup analysis of the different parts of the intracranial arteries. Middle cerebral and internal carotid arteries in the intracranial segments were assigned to the anterior circulation group, and vertebral and basilar arteries in the intracranial segments were assigned to the posterior circulation group. The results revealed a correlation between the QFR and CVPR, which was larger in the anterior circulation. This might be attributed to the different mechanisms in the collateral circulation between the anterior and posterior circulations; in the anterior circulation, the complete circle of Willis is more common than that in the posterior circulation (34, 35). Analysis of different subgroups suggested that QFR and CVPR were moderately correlated at different degrees of stenosis and lesion locations. Non-invasive QFR could better reflect pressure changes in intracranial atherosclerotic stenosis, indirectly indicating whether the lesion was associated with hemodynamic disorders.

Regarding anatomical structure, we analyzed the correlation between CVPR or QFR and the DS% of ICAS. We revealed a negative correlation in individuals with different DS%, and the degree of QFR correlation was slightly higher. This indicates that the more severe the stenosis, the lower the pressure fraction at the distal stenosis, which might imply a greater hemodynamic change in the lesion. From the analysis of the strength of correlation, both r values were below 0.55, suggesting that not all patients with severe stenosis had a lower pressure ratio. It is possible that other factors, such as the influence of collateral circulation within the intracranial arteries, may play a role.

While investigating non-invasive pressure measurement, we also measured RFR without injecting hyperemic drugs. RFR has been recognized and widely used in percutaneous coronary intervention (PCI) guidance for coronary artery stenosis (36, 37). In previous studies on coronary artery diseases, the quantitative value of the RFR was lower than that of the FFR. However, the RFR and CVPR data in our study were in high agreement, which could be because the CVPR data were also measured using non-hyperemic conditions. The calculation methods are based on a coronary artery model. Our team considered that RFR measurements alone could not explain this issue, and compared with CVPR measurements, additional RFR measurements were not necessary. In addition to QFR, other hemodynamic indicators have been established using non-invasive approaches, including computational fluid dynamics modeling and signal intensity ratio based on the quantitative and time-of-flight magnetic resonance angiography (38–42), with promising results. However, all of these have their own limitations. The automatic delineation of artery contours by an artificial intelligence algorithm facilitated by 3D-DSA angiography images might provide more accurate data for intraoperative application. We proposed that the measurement of the direct translesional pressure ratio can potentially have a significant impact. To further investigate this, our neurointerventional team has initiated both a prospective cohort study and a comparative study with quantitative analysis between nuclear magnetic perfusion and CVPR. These studies are expected to provide stronger evidence regarding ICAS. Intracranial pressure measurement of invasive non-hyperemic pressure ratios is a direct pressure value on specific vascular location by using pressure-wire, and the pressure ratio was one of the important indicators of hemodynamics but still could not reflect the reserve function and complete state of cerebral blood flow. The calculation of QFR was in the preliminary phase in cerebrovascular evaluation, and the measured method could be provided a portion of hemodynamic reference value. In the future, artificial intelligence algorithms or algorithms based on 3D-DSA, TCD, and other parameters could be optimized and may be more suitable for intracranial arteries.

### 4.2 Limitations

In this study, we did not perform CTP and PWI examinations, focusing instead on a comparison between invasive pressure guide-wire measurement (CVPR) and non-invasive QFR measurement. Currently, cerebral vascular perfusion examination also plays an important role in clinical treatment decisions. Recognizing that evaluating in ICAS solely by stenosis rate and clinical symptoms is insufficient, the hemodynamics assessment in ICAS was very important, our team is dedicated to studying this condition in more depth. We have on-going research on perfusion-related data and its impact on CVPR, aiming to determine the cut off value of intracranial artery. We expect to have results available soon. Additionally, the small sample size of this study is one of its main limitations, primarily because the pressure guidewire measurement is an invasive procedure and is subject to many constraints. In the future studies, we will live this issue thorough consideration.

## 5 Conclusion

The calculated QFR derived from the angiographic view was related to CVPR by measuring the invasive pressure wire. The repeatability and reliability of QFR in intracranial atherosclerotic stenosis may reflect hemodynamic changes in the cerebral vasculature. Further studies are warranted to demonstrate that CVPR reliably and comprehensively reflects cerebral hemodynamics.

## Data Availability

The original contributions presented in the study are included in the article/supplementary material, further inquiries can be directed to the corresponding author/s.
